# Novel Diagnostic and Therapeutic Approaches to Temporomandibular Dysfunction: A Narrative Review

**DOI:** 10.3390/life13091808

**Published:** 2023-08-25

**Authors:** Brendan Moxley, William Stevens, Joel Sneed, Craig Pearl

**Affiliations:** 1 School of Dentistry, The University of Texas Health Science Center at Houston, Houston, TX 77054, USA; william.t.stevens@uth.tmc.edu (W.S.); joel.p.sneed@uth.tmc.edu (J.S.); 2 Department of Oral and Maxillofacial Surgery, The University of Texas Health Science Center at Houston, Houston, TX 77054, USA; craig.b.pearl@uth.tmc.edu

**Keywords:** novel diagnosis, temporomandibular dysfunction, therapeutic

## Abstract

Temporomandibular dysfunction (TMD) is a burgeoning area of study within the dental field. TMD is caused by abnormalities in the temporomandibular joint or muscles of mastication and can lead to pain, loss of function, and other complications. As this area of patient care receives increased focus, the ability to accurately diagnose TMD becomes paramount. The aim of this review is to summarize novel diagnostic and therapeutic techniques that have been proposed within the last approximately 3 years in order to inform readers of the cutting-edge advances in the field of TMD diagnosis and management, while also analyzing the clinical relevance of each study. A PubMed search was completed on 1 March 2023, using MeSH terms related to TMD diagnosis and treatment. The search yielded seven articles that pertained to the aim of this review article. The main findings from each study are summarized in this review article. These novel methods of diagnosing and treating TMD may improve our ability to assess and treat patients suffering from TMD.

## 1. Introduction

Temporomandibular dysfunction (TMD) is the second most common musculoskeletal disorder that causes pain and disability, affecting nearly 5% of Americans (~16 million people) [1,2,3,4]. TMD is a condition that can be symptom-free, but more often causes patients pain, discomfort, and dysfunction that can be profoundly debilitating. The treatment costs and quality of life burden can be significant on these patients [5].

In fact, the proportion of TMD patients who experience at least one psychological comorbidity is as high as 75% [6]. TMD is caused by dysfunction of the muscles of mastication and/or the temporomandibular joint (TMJ) itself, and can lead to symptoms such as pain, joint noises, impaired jaw function, and locking. This condition can be hard to diagnose, and even harder to treat, as the manifestations of TMD are varied. TMD patients may present overlapping symptoms with other chronic pain conditions, including headache, fibromyalgia, and neurological conditions. The mechanism of this is not certain, but is likely through the phenomenon of central sensitization, such as allodynia and hyperalgesia [7,8,9,10]. There are numerous established methods of diagnosing TMD, although none have a 100% success rate. There are also numerous established therapeutic methods for treating TMD, with more being proposed every year, as this review article will demonstrate; however, no therapeutic method demonstrates a satisfactorily high rate of success.

Currently, the “gold standard” for TMD diagnosis is a Magnetic Resonance Imagine (MRI) scan; however, many patients will suffer from TMD despite no obvious pathological or mechanical disruptions [11]. Thus, TMD can be divided into three groups. Group I includes TMD caused by muscle disorders, including myofascial pain with and without limitations in mouth opening. Group II includes TMD caused by disc displacement with or without reductions and limitations in mouth opening. Group 3 includes arthralgia, arthritis, and arthrosis [12].

As for TMD management, treatment depends on etiology, whether pathological, mechanical, musculoskeletal, or idiopathic. However, in the absence of malignant pathology, conservative management protocols are usually recommended before surgical intervention, which is considered a last-resort treatment. Popular conservative management includes the prescription of muscle relaxants, the use of an occlusal splint, diet modification, and home physiotherapy with patient education [1].

Due in part to the relatively low success rate of existing diagnostic and therapeutic approaches in TMD, novel techniques are constantly being proposed. In this paper, an updated review of the latest techniques is described.

## 2. Materials and Methods

An article by Wu et al. assessed the established and novel therapeutic remedies available to TMD patients up to July of 2020. The aim of this article is to highlight novel diagnostic and therapeutic approaches that have been published since then. In doing so, the PICO question to be addressed is, “for patients suffering from TMD, do the novel diagnostic and therapeutic approaches proposed in the scientific literature since July of 2020 suggest efficacy in accurately diagnosing and managing their TMD compared with established techniques?” We conducted an electronic search of the PubMed database between 1 July 2020 and 1 March 2023 using the MeSH terms “diagnosis and TMD, therapeutic treatment and TMD, new treatment and TMD, new diagnostics and TMD”. In addition to excluding by date, exclusion criteria were papers that were not published in English or those that were not pertinent to TMD diagnosis or treatment (see Table 1).

## 3. Results

**Figure 1:**
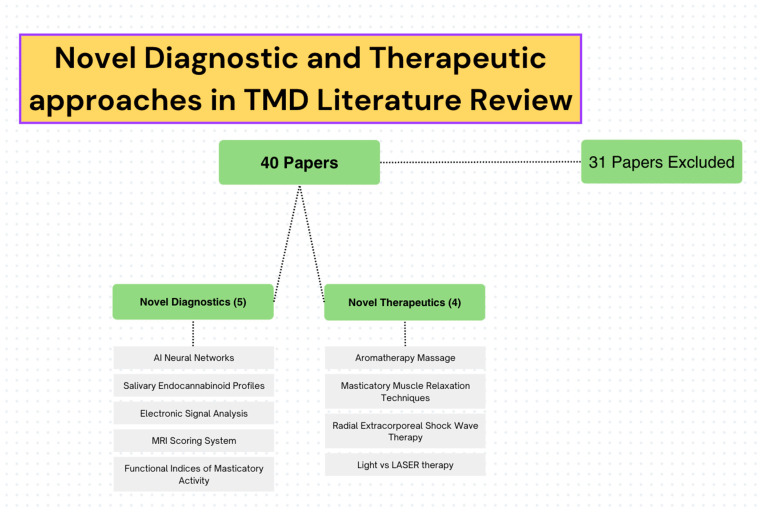
A graphical representation of the literature review conducted.

In total, 40 results were returned upon conducting the PubMed search, as described above (see Figure 1). Five papers on novel TMD diagnosis were analyzed as fitting the criteria for this paper. Additionally, four papers on novel TMD therapeutics were analyzed as fitting the criteria for this study. The topics of each of the papers on TMD diagnosis were AI neural networks, salivary endocannabinoid profiles, electronic signal analysis, a novel MRI scoring system for TMD, and novel functional indices of masticatory activity. The topics of the papers relating to TMD therapeutics include aromatherapy massage, masticatory muscle relaxation techniques, radial extracorporeal shock wave therapy, and light and LASER therapies (see Figure 1).

### 3.1. Novel Diagnostic Approaches to Temporomandibular Dysfunction

#### 3.1.1. Novel Artificial Neural Network for TMD Diagnosis

At this time, artificial intelligence (AI) is primarily used within the medical field to aid in the diagnosis and treatment of life-threatening conditions such as cardiovascular disease and cancer. Although TMD has a similar prevalence to these conditions in the general population, the use of artificial intelligence for the diagnosis and treatment of TMD is still in its infancy. While TMD is not itself a life-threatening condition, TMD can present with similar symptoms to life-threatening conditions. In fact, 4% of acute myocardial infarctions present with pain in the craniofacial structures as the only symptom [13]. The complexity of diagnosing TMD, in conjunction with the fact that many orofacial pain symptoms arise from other parts of the body, make proper TMD diagnosis a significant challenge for the general practitioner.

Given the successful use of AI for the diagnosis and treatment of other areas of medicine, Kreiner et al. attempted to develop an algorithm to diagnose orofacial pain and TMD with equal or higher accuracy than a general dentist [13]. Kreiner et al. created an artificial neural network (ANN) which is a subset of AI. This program allows the input of multiple pieces of information (patient symptoms, diagnostic imaging, etc.) and creates an output (diagnosis). The Kreiner ANN was given the same patient scenarios as 12 general dentists and asked to determine whether the patient’s pain was of orofacial origin, and if so, to properly diagnose the condition. The neural network outperformed the dentists on average. The neural network was clearly superior at diagnosing pain from outside the orofacial region (e.g., referred cardiac pain, neuropathic pain). For example, only 25% of clinicians could diagnose the cases of referred orofacial pain from myocardial infarctions. The clinicians frequently chose diagnoses of “occlusal trauma”, “bruxism”, “periodontal disease”, and “I do not know” instead. Only two clinicians could accurately diagnose the patient suffering from migraine symptoms. Only half of the clinicians correctly diagnosed TMD, while the ANN was able to correctly identify all cases. For pain of odontogenic origin (e.g., pulpitis), there was no significant difference between the ANN and the general dentists.

This study found that the novel ANN showed high diagnostic accuracy for the diagnosis of TMD. The results showed that the ANN had a sensitivity of 96.9% and a specificity of 95.5% for the diagnosis of TMD. These results indicate that the ANN was highly accurate in identifying patients with TMD and distinguishing them from patients without TMD. This level of accuracy was found to be comparable to or better than other diagnostic tools currently used for TMD diagnosis, such as clinical examination and imaging.

The results suggest that the ANN could be a useful tool for the diagnosis of orofacial pain and TMD. The ANN has several advantages over traditional diagnostic tools, including the ability to quickly and accurately analyze large amounts of data and identify patterns and relationships that might be missed by human observers. Further research could focus on improving the ANN’s diagnostic algorithms and exploring other potential applications for ANN technology in dentistry and medicine.

Overall, this study demonstrates the promise of novel diagnostic tools using artificial intelligence. A simple neural network with only five layers of coding was able to outperform a dozen clinicians on diagnoses ranging from TMD to migraines to referred cardiac pain. More testing is needed before drawing conclusions about the widespread applicability of this ANN, and more AI algorithms like it. However, this is an area that contains vast potential for simplifying and streamlining the otherwise complicated task of accurately diagnosing patients with TMD.

#### 3.1.2. Salivary Endocannabinoid Profiles

Each individual possesses a unique endocannabinoid (eCB) profile within their saliva. It has been suggested that this eCB profile may indicate the presence of underlying conditions that cause pain, such as temporomandibular disorder. The eCB profile of patients diagnosed with certain orofacial pain disorders has not been thoroughly studied. A study by Heiliczer et al. attempted to classify certain eCB profiles according to patients with current diagnoses of post-traumatic neuropathy, trigeminal neuralgia, temporomandibular disorders, migraine, tension-type headaches, and burning mouth syndrome [14]. Correlation analyses between eCB levels, a current and specific diagnosis, and pain characteristics were conducted.

**Figure 2:**
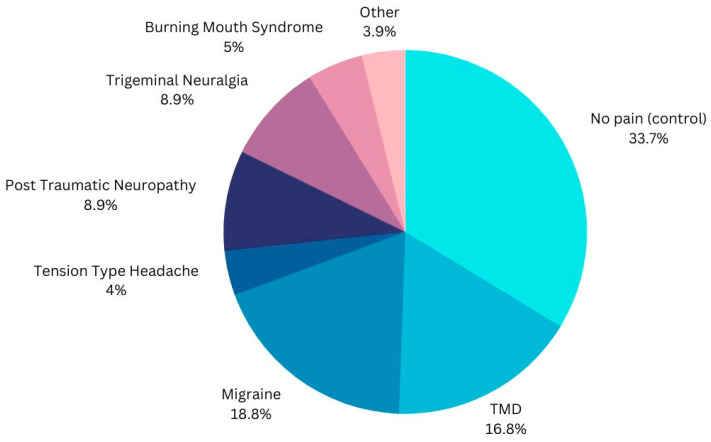
The percentages of each of subcategories of the 126 participants are displayed.

The study enrolled 126 participants, including 83 patients with chronic orofacial pain or headache disorders and 43 healthy controls (see Figure 2). Saliva samples were collected from all participants, and salivary levels of endocannabinoids and related compounds were analyzed using liquid chromatography–tandem mass spectrometry. The results showed significant differences in salivary endocannabinoid profiles between the patient group and the control group. Specifically, the patient group exhibited significantly lower levels of anandamide and 2-arachidonoylglycerol (AG), two endocannabinoids with known analgesic and anti-inflammatory properties. The group of patients who currently suffer from migraines had significantly lower levels of an eCB called PEA in their saliva. There was a significantly increased level of AEA in the saliva of patients who suffer from burning mouth syndrome. There were not significantly increased or decreased levels of any specific eCBs in the temporomandibular disorder or post-traumatic neuropathy groups of this study.

Heiliczer et al. suggest that these findings could have several clinical implications. For example, salivary endocannabinoid profiles could be used as a diagnostic tool for chronic orofacial pain and headache disorders. Additionally, the findings suggest that endocannabinoid-based therapies, such as cannabinoid receptor agonists or inhibitors of endocannabinoid degradation, could be effective in treating these conditions. Heiliczer et al. also noted several limitations of the study, including the relatively small sample size and the lack of longitudinal data. Future studies could address these limitations by enrolling larger cohorts and following patients over time to assess the long-term effectiveness of endocannabinoid-based therapies.

Overall, the study suggests that salivary endocannabinoid profiles could be a useful tool for the diagnosis and management of chronic orofacial pain and headache disorders. The findings also support the potential therapeutic value of endocannabinoid-based therapies in these conditions. Further research is needed to confirm these findings and to explore the underlying mechanisms of endocannabinoid signaling in chronic pain and headache disorders. The fact that salivary samples of patients with certain orofacial pain disorders demonstrated signature eCB patterns suggests that more research should be conducted in this field. The potential to elucidate a certain eCB marker that correlates with temporomandibular disorder exists and would aid in the diagnosis of TMD patients going forward.

#### 3.1.3. Signal Analysis to Diagnose TMJ Hypermobility

Frequently, certain noises emanating from the temporomandibular joint (TMJ) lead patients and clinicians to assume that temporomandibular dysfunction (TMD) must be present. These noises often including clicking, popping, and crepitus, among others. These noises can occur during speaking, eating, yawning, and other daily activities. However, these noises do not always coincide with disorders, and can lead to misdiagnoses. In fact, the noises caused by TMJ hypermobility are often errantly assumed to be caused by TMD [15]. Grochala et al. conducted a study of noises emanating from the TMJ using a novel technique called signal analysis [16]. This is a non-invasive technique that uses an electronic stethoscope to record noises associated with the TMJ during the process of opening and closing.

The researchers enrolled 47 patients who experienced noises emanating from their temporomandibular joints. The participants completed the official and commonly used research diagnostic criteria for temporomandibular disorder (RDC/TMD) questionnaire to determine whether they likely had TMD or TMJ hypermobility. Next, a Littmann 3200 electronic stethoscope was used simultaneously on both sides of the head to record TMJ sounds in function. These recordings were transferred to a computer for analysis of the signals produced during opening and closing. The research revealed time–frequency features in acoustic signals that are characteristic to TMJ hypermobility.

In this study, signal analysis was used to identify characteristic features of TMJ hypermobility, such as abnormal movement patterns and increased joint laxity. Grochala et al. developed a set of signal processing algorithms that were able to accurately distinguish between patients with TMJ hypermobility and control subjects based on these features. The signals were analyzed using several signal processing techniques, including Fourier transform, Hilbert transform, and wavelet transform. These techniques enable the decomposition of complex signals into their component frequencies and amplitudes, which can then be analyzed to identify patterns and anomalies.

The results showed that the new diagnostic method was able to accurately distinguish between patients with TMJ hypermobility and control subjects. Specifically, the method had a sensitivity of 98.8% and a specificity of 100%, indicating that it was highly accurate in identifying patients with TMJ hypermobility and excluding individuals without the condition. The authors suggest that this novel diagnostic method could have several clinical implications. For example, it could be used to identify patients with TMJ hypermobility who might be at risk of developing more severe TMJ disorders or who might benefit from early intervention to prevent further damage. Additionally, the method could be used to monitor the effectiveness of TMJ treatments over time. The authors note several limitations of the study, including the relatively small sample size and the lack of validation in other patient populations. Further research is needed to confirm the diagnostic accuracy of this new method and to assess its potential clinical utility.

Signal analysis is a powerful tool for diagnosing and monitoring TMJ disorders because it enables the objective measurement of joint movement and function. This can be especially useful in cases where clinical examination alone may be insufficient to diagnose or monitor the progression of the condition. Additionally, signal analysis can provide valuable insights into the underlying biomechanical mechanisms of TMJ disorders, which can inform the development of new treatments and interventions. By creating a database of certain signals unique to patients with TMD versus those with TMJ hypermobility, practitioners may be able to compare the sounds of undiagnosed patients with the signals unique to certain diagnoses, and more accurately diagnose new patients. This is an area of research that still needs extensive study and data collection before it can be definitively used as a diagnostic aid; however, the potential benefit exists.

#### 3.1.4. A Novel MRI Scoring System for TMD Diagnosis

Of the many diagnostic tools used by clinicians to diagnose TMD, magnetic resonance imaging (MRI) is widely considered the gold standard [17]. Not only does this imaging modality confer a high level of resolution of hard and soft tissue structures in the TMJ, but it can also produce imaging of the joint in motion. However, MRI is only useful for diagnosis if there is a reliable system for assessing the imaging and relating it to a diagnosis. In 2018, Wurm et al. proposed a novel MRI-based scoring system to diagnose TMD. This system offers a standardized evaluation using three main variables that assess key relevant structural changes within the TMJ [18]. This system includes an assessment of the articular disc, the direction of disc luxation, and osseous joint alterations. Although this novel system has potential to be a useful diagnostic tool, the inter-rater reliability of the system has not been assessed.

In 2022, Willenbrock et al. conducted a study to assess the inter- and intra-rater reliability of the Wurm et al. MRI scoring system [19]. Willenbrock et al. enrolled 60 patients with suspected uni- and bilateral TMD for assessment by two experienced radiologists. Using MRI of these patients’ TMJs, the radiologists scored each patient on the Wurm et al. scoring system. Inter-rater and intra-rater reliability were assessed using two different methods: intraclass correlation coefficient (ICC) and weighted kappa coefficient. The results showed high reliability for both methods, with ICC values ranging from 0.85 to 0.99 and kappa values ranging from 0.76 to 1.00. No significant differences were found between both observers for the articular disc and direction of disc luxation scores. Although significant differences were found for the assessment of subtle osseous changes, these differences were minor.

The correlation between the MRI-based scores and clinical assessments of TMD severity was also evaluated. The clinical assessments included pain intensity, jaw opening, and joint sounds. The results showed a significant correlation between the MRI-based scores and all three clinical assessments, indicating that the scoring system was able to accurately reflect the severity of TMD. Willenbrock et al. noted that the new scoring system has several advantages over existing systems. For example, it is based on MRI, which enables the non-invasive assessment of TMJ abnormalities. Additionally, the system includes multiple categories of abnormalities, which provides a more comprehensive assessment of TMJ health. However, Willenbrock et al. also noted some limitations of the system. For example, the system requires specialized training to use, which may limit its accessibility to some clinicians. Additionally, the system may not be able to detect some types of TMJ abnormalities that are not visible on MRI scans.

In conclusion, Willenbrock et al.’s study concluded that the novel Wurm et al. scoring system is reliable enough to use as a tool in the process of diagnosing patients with suspected TMD. The high reliability and validity of the system suggest that it could be a useful tool for clinicians in diagnosing and managing TMDs. Further research is needed to validate the system in larger patient populations and to explore its potential clinical applications.

#### 3.1.5. Novel Functional Indices of Masticatory Muscle Activity

It is well known that for muscle activity to occur, specific electrical signal pathways within the musculature must occur. This includes the muscles surrounding the TMJ during functional movements and at rest. The practice of measuring electromyography has been applied to diagnosing bruxism [20], tension-type headaches [21], Down syndrome [22], different occlusal features [23], motor neuron disease [24], and in TMD patients [25]. Ginszt et al. hypothesized that analyzing masticatory muscle activity in patients with signs of TMD using novel functional indices could aid in more accurate diagnoses [26]. This team conducted a study in which 78 women were divided equally into two groups based on an existing diagnosis of TMD or a healthy adult. In order to record the bioelectrical activity of facial musculature, surface electromyography was used. The bioelectric activity of the temporalis anterior, the superficial masseter, and anterior bellies of the digastric muscles was recorded during functional clenching, functional opening, opening, and at rest. The data collected were analyzed using a wavelet transformer to extract the time–frequency characteristics of the surface electromyography signals. Ginszt et al. developed three functional indices: the muscle activation index, muscle activity rhythm index, and muscle contraction force index.

Statistical analysis of the results demonstrated significant differences between the control group and TMD group in various categories. The results showed that the muscle activation index was highest during clenching and lowest during chewing soft food. The muscle activity rhythm index was highest during chewing hard food and lowest during clenching. The muscle contraction force index was highest during chewing hard food and lowest during chewing soft food. It was found that the control group had higher values in all functional clenching indices. The most significant difference occurred on the left side during clenching activity. It was found that there was considerable difference within the range of motion during maximum active mouth opening. There were also significant differences in some measurements in the resting position.

Although this study yielded statistically significant results between TMD and non-TMD patients, the applicability of these results is still unreliable. Measuring electromyographic activity is a complex process, and the results cannot be predictably applied to patient diagnoses and treatment recommendations at this time. There is potential for further research in this area that could elucidate specific diagnosable trigger points and muscle activity particular to TMD patients. Ginszt et al. posits that in order to verify and confirm the validity and effectiveness of the use of the functional indices, replication studies must be performed.

### 3.2. Novel Therapeutic Approaches to Temporomandibular Dysfunction

A PubMed search using the inclusion and exclusion criteria described in the Introduction yielded very few results for novel therapeutic tools published since July 2020. In total, four papers will be reviewed, but the first two articles described below are of limited applicability, relevance, and/or reliability. They have been included in this review article for the purpose of completeness.

#### Novel TMD Treatment Using Aromatherapy Massage with Lavender Oil

A study was published in the Journal of Craniofacial and Sleep Practice that investigated the effects of massage therapy on alleviating TMD pain symptoms. This therapy theoretically reduces pain via activation of the pain–gate pathway, stimulates the parasympathetic center, and re-establishes muscular length and flexibility, improves local blood circulation, and increases the production of endogenous opioids [27]. This area of study has not received extensive study, and there is not a large body of evidence supporting its efficacy.

Benli et al. conducted a randomized controlled trial to investigate the effects of aromatherapy massage with lavender oil on pain reduction and maximal mouth opening in patients with myogenous TMD compared with a control group. For this study, 90 patients were selected based on stringent eligibility criteria: 30 patients were placed in the test group, which received aromatherapy massage with lavender oil; 30 patients were placed in the placebo group, and received massage therapy with sweet almond oil; 30 patients were placed in the control group, and received no massage therapy. All patients abstained from taking analgesic medication during the trial. Efforts were made to adequately control for other variables; however, a detailed description of those measures is outside the scope of this review.

The findings indicated that the aromatherapy massage group showed significant differences compared with control and placebo groups in terms of maximal mouth opening and the evaluated pain parameters (as measured by visual analog scale). At the beginning of the trial, there was no difference in the two measurements between all groups. Immediately after the treatment, both groups that received aromatherapy massage demonstrated statistically significant improvements in pain reduction and maximal opening compared with the control group. The group that received lavender oil treatment demonstrated significant improvements compared with the placebo group that received almond oil. At two months post-procedure, both massage groups again demonstrated significant improvements compared with the control group. Similarly, the group that received lavender oil demonstrated more pain reduction and greater maximal opening than the almond oil group. However, the difference in results compared with the control group declined compared with measurements taken immediately after treatment. The limited results seemed to suggest that the beneficial effects of treatment waned after a short period of time. These results suggest that there may be merit to further investigation into the use of both massage therapy and use of lavender oil as adjunctive, conservative treatment for pain reduction and increased maximal opening in TMD patients.

### 3.3. Novel Manual Techniques in Masticatory Muscle Relaxation in TMD Treatment

A study was published in the International Journal of Environmental Research and Public Health that investigates the degree of relaxation of muscles of mastication achieved by manual release techniques. This study by Urbański et al. enrolled patients who are currently undergoing prosthetic treatment to relieve TMD with a dominant muscular component [28]. Sixty patients were randomly assigned to a group that received post-isometric relaxation treatment or a group that received myofascial release treatment. Both groups received ten treatment sessions and were assessed using surface electromyography measurements of the anterior temporal and masseter muscles as well as the intensity of spontaneous masticatory muscle pain assessed via the visual analog scale.

The results from the study demonstrated that both groups exhibited decreased electrical activity in the temporalis and masseter muscles after treatment. Both treatment groups also exhibited a significant drop in the intensity of spontaneous pain in the masticatory muscle group. There was no significant difference in results between the two treatment groups. Urbański et al. suggest that both post-isometric relaxation treatment and myofascial release treatment are appropriate adjunctive treatments for TMD patients receiving prosthetic treatment. The authors also discussed the mechanisms by which manual techniques may exert their therapeutic effects. They suggested that manual techniques may help to release tension and adhesions in the masticatory muscles, increase blood flow and oxygen supply to the muscles, and stimulate the release of endorphins and other pain-relieving substances.

Overall, Urbański et al. concluded that manual techniques can be a valuable adjunctive therapy in the treatment of TMDs. They suggested that manual techniques should be considered as part of a comprehensive treatment plan that includes patient education, stress management, and other therapies, such as physical therapy and pharmacological interventions. While this area of treatment demonstrates potential for certain patients, more research is recommended before definitively adopting this treatment modality for TMD patients.

#### 3.3.1. Radial Extracorporeal Shock Wave Therapy for TMD

Radial Extracorporeal Shock Wave Therapy (rESWT) is an established treatment modality for treating a variety of musculoskeletal conditions. It involves using a machine with a tip that appears similar to an ultrasound. It administers shock waves to the painful, tense area for approximately 3 min every week, and has been shown to effectively reduce pain in the area over time. In this study, this technique was tested in patients with temporomandibular disorders to determine its efficacy in treating the pain exhibited by these patients.

The study design included two groups: the first with eight patients who underwent a series of physical exercises combined with rESWT, and the second group of seven patients who served as a control as they underwent the same series of exercises but with sham rESWT. The treatment regiment included 20 min of bilateral manual physical therapy followed by 3 min of rESWT, one session a week for four weeks. Efficacy was measured using two data points: the patient’s self-reported pain intensity according to the visual analogue scale (VAS), as well as a surface electromyography evaluation (sEMG) of the anterior temporalis and masseter muscles to assess muscle function. The sEMG test was performed using four surface electrodes to detect the electrical activity of the masticatory muscles. As these patients presented with trigger points, reduced electrical activity was desired in the post-test for these patients as compared with the pre-test [29].

The results of the study were that the rESWT group exhibited statistically significant pain reduction compared with the group receiving sham rESWT. Additionally, while some providers question the efficacy of the sEMG test in clinical use, there were several statistically significant data points between the two groups for this test as well. The authors state that, to the best of their knowledge, this is the first study on rESWT in this application, so further studies are warranted to confirm the efficacy of these findings, especially since this technique demonstrates conflicting evidence in other areas of the body [30].

#### 3.3.2. Light Therapy vs. LASER in Pain Reduction in TMD

Both red light therapy and LASER are techniques for treating TMD that have been shown to be effective when compared with a control group in previous studies; however, this study compared the two techniques to each other due to their similar mechanism of action [31].

In terms of the mechanism of action, red light therapy works by providing heat to the tender area, which causes vasodilatation of the adjacent blood vessels and increased blood supply. The increased blood supply washes away the inflammatory mediators, which improves patient symptoms. The mechanism of action of LASER therapy is similar, although it is theorized that LASER therapy also increases cell metabolism and protein synthesis. The biggest difference between the mechanism of the two techniques, however, is the specific wavelength of the energy delivered, with LASER being higher energy that is administered for a shorter duration.

Patients were randomly assigned to one of three groups: group A served as the control because the LED light device was applied to the trigger points without turning them on; group B received the LED light device that was turned on for 5 min; and group C received low-level LASER therapy for 30 s. The VAS pain score and presence of trismus and trigger points was assessed in four visits over 4 weeks.

The results of the study are that patients receiving either light therapy or LASER showed statistically significant improvements in pain when compared with a control (consistent with previously published literature); however, there were no differences between LASER or light therapy groups when comparing the two treatments with each other. Regarding tenderness, both techniques were effective in reducing the number of trigger points, but the LASER technique was statistically significant, while the red light therapy was insignificant. However, LASER devices are more expensive and more invasive, so the lower cost and higher biosafety of the LED device may make it more clinically viable as a technique [32,33].

## 4. Discussion

The field of TMD diagnosis and treatment is rapidly developing as our understanding of the different etiologies of TMD progresses. Although numerous diagnostic and treatment modalities have been established, room exists for improvement and innovation in this area. Since July 2020, five novel diagnostic modalities and two novel therapeutic methods have been proposed. The novel diagnostic modalities of an artificial neural network for TMD diagnosis demonstrated the ability to diagnosis TMD and other myofascial pain syndromes with equal or superior accuracy compared with clinicians. The use of salivary endocannabinoid profiles demonstrated potential as a method to non-invasively screen patients for TMD and other conditions that elicit distinct endocannabinoid profiles. The use of signal analysis to diagnose TMJ hypermobility could potentially reduce the number of patients who are erroneously diagnosed with TMD when, in fact, they have hypermobile temporomandibular joints. The repeated testing of a novel MRI scoring system demonstrated efficacy of the system and lends support to the continued use of it as part of the TMD diagnosis algorithm. Finally, the use of novel functional indices of masticatory muscle activity demonstrates potential as an adjunctive tool for accurate TMD diagnosis.

Furthermore, since July 2020, four novel therapeutic methods have been proposed. The use of aromatherapy massage with lavender oil as a treatment modality for TMD demonstrated positive results. The use of manual techniques in masticatory muscle relaxation to treat TMD also demonstrated potential as an adjunctive treatment modality. However, these studies were limited by small sample sizes and reduced follow-up, so further research is indicated before these therapeutic modalities are adopted as standard treatments. Radial extracorporeal shock wave therapy, red light therapy, and LASER therapy are not new treatment modalities for musculoskeletal problems generally, but studies published within the last 3 years suggest efficacy for managing TMD.

Gender and age were not defined as exclusion criteria for this narrative review; however, TMD has been shown to present in higher frequency and severity in females than males, with peak severity between ages of 20 and 40, which is consistent with the demographic data of the patients enrolled in each study reviewed [34,35,36,37].

Although some studies confer more evidence than others, these studies represent the expanding boundaries of this area of patient care. There were limitations to the scope of this review. In some cases, the same novel approach had multiple publications within the last three years, especially in the topic of artificial intelligence (AI) for TMD diagnosis [35,36,37]. Additionally, although best efforts were undertaken to analyze all novel diagnostic and therapeutic approaches to temporomandibular dysfunction, there are likely some research articles that were not included.

In terms of clinical utility, further studies are warranted for all described novel diagnostic and therapeutic approaches before clinical adoption is recommended. The only therapeutic approaches that are potentially clinically relevant at the current stage are radial extracorporeal shock wave therapy and red light or LASER therapy due to the abundant literature on these techniques in other parts of the body confirming their safety and efficacy, combined with preliminary evidence suggesting that this safety and efficacy extends to the temporomandibular region as well. However, all approaches are potentially suitable in the academic setting if tested as the subject of further investigation.

## 5. Conclusions

Temporomandibular dysfunction is a condition that affects a significant proportion of the population. TMD carries societal cost burdens as well as a substantially reduced quality of life for many patients. The articles included in this review represent the boundaries that are being expanded in an effort to better care for this patient population. Although this field has achieved considerable progress in recent years, further research is recommended to advance the care of patients suffering from TMD.

## Figures and Tables

**Table 1:** Inclusion and exclusion criteria for the narrative review.

Inclusion Criteria	Exclusion Criteria
MeSH terms “diagnosis and TMD, therapeutic treatment and TMD, new treatment and TMD, new diagnostics and TMD”	Papers not pertinent to TMD Diagnosis or treatment
Published between 1 July 2020 and 1 March 2023	Papers not published in English

## Data Availability

Not applicable.
